# Lemierre syndrome: case presentation of a life-threatening septic pneumonia with complicated parapneumonic effusion: A case report

**DOI:** 10.1097/MD.0000000000041102

**Published:** 2024-12-27

**Authors:** Tae Hun Kim, Seong Hwan Youn, Mi-Ae Kim, Hyun Jung Kim, Yong Shik Kwon, Jae Seok Park, Sun Hyo Park

**Affiliations:** a Division of Pulmonary Medicine, Department of Internal Medicine, Keimyung University School of Medicine, Dongsan Hospital, Daegu, South Korea.

**Keywords:** alteplase, Lemierre syndrome, parapneumonic effusion, respiratory failure, sepsis, septic emboli

## Abstract

**Rationale:**

Lemierre syndrome is a rare, life-threatening complication of oropharyngeal infections.

**Patient concerns:**

A 35-year-old man started with an upper respiratory infection but worsened the clinical course with sepsis and acute respiratory failure with complicated bilateral pleural effusion.

**Diagnoses:**

The patient was diagnosed with typical Lemierre syndrome with lung complications.

**Interventions:**

Antibiotic therapy with bilateral pleural percutaneous drain with fibrinolysis.

**Outcomes:**

The patient improved and was discharged without oxygen therapy after antibiotics were covered and active lung care with complicated parapneumonic effusion. After discharge, lung function showed restrictive lung defect but improved compared to the initial exam.

**Lessons:**

Lemierre syndrome, which might begin as a mild upper respiratory infection, can progress to a critically ill disease accompanied by sepsis and metastatic septic embolus. The patient suffered septic lung emboli with bilateral complicated parapneumonic effusion but was successfully treated with percutaneous drainage with pleural fibrinolysis, appropriate antibiotics, and anticoagulants. Early suspicion of the disease and active treatment are necessary to treat rare syndromes like Lemierre syndrome.

## 1. Introduction

Lemierre syndrome is a rare, life-threatening complication of oropharyngeal infections. Lemierre^[1]^ reported 20 cases of sepsis due to anaerobic pathogens.^[1]^ Lemierre syndrome typically affects previously healthy young adults. A previous review reported that patients had a median age of 21 years and 61% were male.^[2]^ The mortality rate significantly decreased to about 2% to 4% after antibiotics were developed in the 21st century.^[2–4]^

## 2. Case presentation

The patient was a 35-year-old male who visited the emergency room with general myalgia, neck pain, and fever (40°C), and the onset of the disease 6 days ago with a sore throat, cough, and rhinorrhea. The patient was treated with symptomatic therapy with over-the-counter medicine. However, neck pain and myalgia continued to worsen. The patient had no previous illness such as hypertension or diabetes mellitus. He was a current smoker with 10 pack-years of smoking history but denied consumption of alcohol.

**Figure 1.**
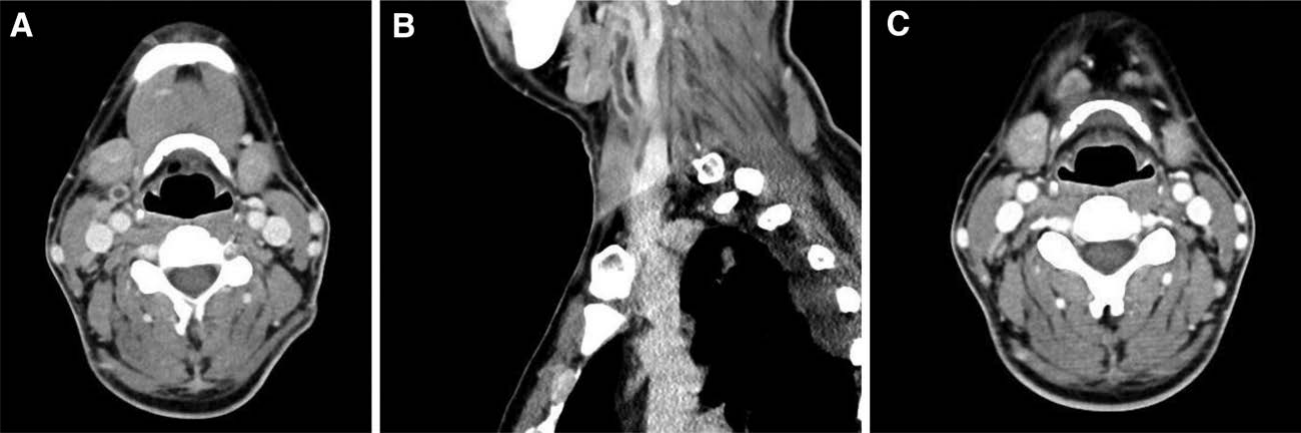
(A, B) Initial neck computed tomography showing a thrombus in the internal jugular vein. (C) Follow-up chest computed tomography on day 13. Neck computed tomography showing resolved thrombus.

**Figure 2.**
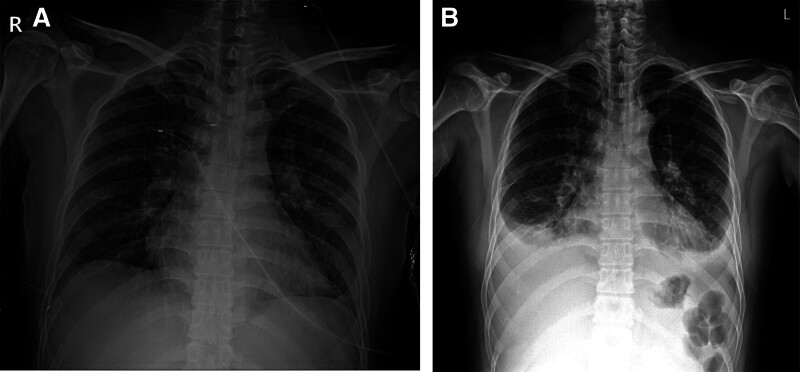
(A) Initial chest radiography. (B) Chest radiography at the time of patient disorientation.

**Figure 3.**
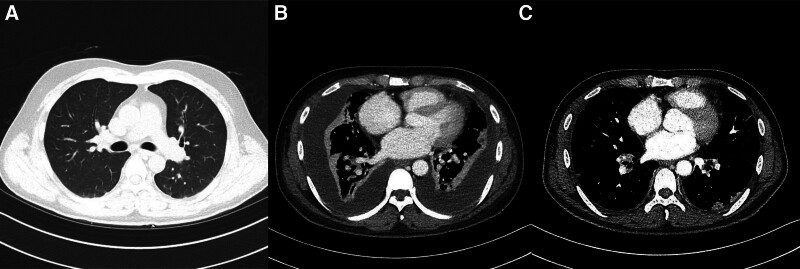
(A) Initial chest computed tomography without active lung lesions. (B) Chest computed tomography at the time of patient disorientation with bilateral pleural effusion and lung nodules. (C) Chest computed tomography after critical care.

First, the patient was admitted to the otolaryngology department for thrombophlebitis at the branch of the right internal jugular vein (Fig. 1A and B) and multiple significant lymph node enlargement was present at right levels II and III. At the time of admission to the hospital, the patient had a fever (39.8°C), tachycardia (140 bpm), blood pressure of 100/50 mm Hg, and normal oxygen saturation (99%) at room air. On physical examination, no external neck erythema and swelling were visible but there was focal tenderness in the right cervical region. The throat was clear and on laryngoscopy, and there were clear and well-mobile true vocal cords. Chest examination revealed clear breathing sounds without wheezing and a heart murmur. There were no neurological abnormalities. Initial laboratory tests are shown in Table 1; radiography showed diffuse peribranchial infiltration with subsegmental atelectasis (Fig. 2A), computed tomography (CT) showed no definite acute lung parenchymal lesion (Fig. 3A), and blood cultures were obtained from 2 bottles each for aerobic and anaerobic organisms. The patient was treated with piperacillin/tazobactam (4.5 mg/6 h) and clindamycin (900 mg/8 h). After 5 days of treatment, the clinical course worsened. The oxygen supply was increased to 6 L using a nasal cannula to achieve a saturation of 90%, and chest radiography findings also worsened. CT was performed, and the patient was moved to the intensive care unit and referred to the department of internal medicine, division of pulmonary medicine. A high-flow nasal cannula with a fraction of inspired oxygen of 0.6 was used, and oxygen saturation was maintained at 92%. Bilateral pleural effusion was present on chest radiography (Fig. 2B). CT showed bilateral pleural effusion with multiple lung parenchymal nodules (Fig. 3B). Antibiotic treatment was considered to have failed, and the regimen was changed to meropenem (1000 mg/8 h, prolonged infusion over 4 hours) with vancomycin (1000 mg/12 h, 15 mg/kg; the drug trough level was checked every 3 days and the dose was adjusted). Percutaneous drain (PCD) catheters were also applied to both pleural spaces. The effusion study showed complicated parapneumonic effusion (right lung: white blood cells 456 cells/μL with 60% neutrophils, lactate dehydrogenase 2400 U/L, protein 3.6 g/dL, glucose 101 mg/dL, pH 7.82; left lung: white blood cells 802 cells/μL with 85% neutrophils, lactate dehydrogenase 1778 U/L, protein 4.2 g/dL, glucose 97 mg/dL, pH 7.36). In the microbiology evaluation, although pleural effusion cultures were obtained after the administration of antibiotics, no pathogen was detected. The patient was subsequently started on a therapeutic dose of low-molecular-weight heparin. As the effusion drain was insufficient, after 2 days of natural drainage, alteplase was injected into both PCDs with the following protocol: alteplase 10 mg mixed with 30 mL of 0.9% normal saline 30 mL; 10 mL of 0.9% normal saline was administered to the PCD; clamping the PCD over 4 hours; and negative regulator applied overnight. After pleural fibrinolytic therapy and sepsis treatment, the patient was improving, and the oxygen demand necessary to maintain a saturation of 92% decreased to 2 L administered through a nasal cannula.

**Table 1:** Results of baseline laboratory test.

Laboratory test	Value	Reference range
White blood cells (10^3^/μL)	10.06	4.00–10.00
Neutrophils (%)	93.70	38.40–73.00
Hemoglobin (g/dL)	12.00	13.00–17.00
Platelets (10^3^/μL)	72.00	130.00–400.00
Erythrocyte sedimentation rate (mm/h)	120	0–15
C-reactive protein (mg/dL)	29.8	0.0–0.5
Procalcitonin (ng/mL)	>100.000	0.000–0.046
Prothrombin time (s)	11.8	10.0–14.0
Activated partial thromboplastin time (s)	26.8	20.0–33.5
Fibrinogen (mg/dL)	1162.5	200.0–400.0
D-dimer (μg/mL)	1.97	<0.40
Total bilirubin (mg/dL)	4.02	0.2–1.2
Direct bilirubin (mg/dL)	3.29	0.0–0.42
Albumin (g/dL)	3.3	3.5–5.2
Blood urea nitrogen (mg/dL)	26	6–20
Creatinine (mg/dL)	1.20	0.7–1.2
Lactate (mmol/L)	1.3	0.4–0.8

The blood culture revealed the presence of *Fusobacterium necrophorum*. Sequential culturing was obtained after 2 days of piperacillin/tazobactam and clindamycin administration, but culture-negative conversion was not achieved. The presence of methicillin-resistant staphylococcus aureus was confirmed on sputum cultures. During the administration of meropenem and vancomycin, the fever subsided steadily, and the oxygen demand also improved. One week after the transfer to the intensive care unit, oxygen demands were improving but the patient experienced a drug eruption; thus, vancomycin was withdrawn. Two weeks after the transfer, a follow-up blood culture confirmed negative conversion. CT showed resolution of the venous thrombosis of the right jugular vein (Fig. 1C) and pleural effusion, and multiple septic nodules decreased in number and size (right upper lung 20 mm, 14 mm nodule was improved to 8 mm, and 6 mm, respectively) (Fig. 3C). After 4 weeks of antibiotic treatment, shortness of breath and pleuritic chest pain (visual analog scale 3) were still present, but there was no hypoxemia even without oxygen therapy. Spirometry showed restrictive lung disease. After intensive care, follow-up at the outpatient department revealed a significant improvement in the shortness of breath and that the pleuritic chest pain had resolved. Lung function tests had also improved (Table 2).

**Table 2:** Results of the spirometry and the pulmonary function test.

	Immediately after intensive care treatment	Three-month follow-up
FEV1/FVC	0.98	0.96
FEV1 (% of predicted)	51	76
FEV1 (L)	1.93	2.87
FVC (% of predicted)	41	62
FVC (L)	1.97	2.97
TLC (% of predicted)		67
RV (% of predicted)		68
DL_CO_ (% of predicted)		65

DL_CO_ = diffusion capacity of carbon monoxide, FEV1 = forced expiratory volume in 1 s; FVC = forced vital capacity, RV = residual volume, TLC = total lung capacity.

## 3. Discussion

Lemierre^[1]^ reported on 20 patients (18 of which died) with combined anaerobic and septic bacterial infection after tonsilitis. Since then, several studies have reported on similar cases.^[5–9]^ *F necrophorum*, an anaerobic gram-negative bacillus present in the normal oral flora, is the most common cause of Lemierre syndrome and is isolated in 48% to 82% of cases.^[10–15]^ The causative microbe enters through the mucosa. The pathogen spreads through deep cervical tissue into the hematogenous or lymphatic drain and progresses to the veins, most commonly to the internal jugular vein. Although a general agreement on key clinical features of Lemierre syndrome has been achieved, there is currently no consensus on the definition. One suggested set of criteria includes history of oropharyngeal infection in the last 4 weeks; metastatic embolic infection to end organs; evidence of internal jugular vein thrombophlebitis; and isolation of *F necrophorum* from blood cultures.^[12]^ Complications could develop due to septic emboli on the lungs, joints, brain, and liver.^[2]^ The lungs are a commonly affected site, and patients may develop pneumonia, empyema, pleural effusion, or septic embolus-induced pulmonary infarction.^[16,17]^

The present case experienced severe pneumonia with respiratory failure and complicated pleural effusion. Practical guidelines recommend combination tissue plasminogen activator and DNAse in cases where initial drainage has ceased^[18]^ and state that single-agent tissue plasminogen activator or DNAse should not be considered for treatment.^[18]^ However, DNAse was not available at our institution for intrapleural infusion, making this a non-feasible option. Although we considered decortication surgery, the patient recovered. The treatment of Lemierre syndrome requires a multidisciplinary approach.^[19]^ Antibiotics are the main treatment, and beta-lactams or carbapenems in combination with anti-anaerobe antibiotics are commonly used.^[20]^ *F necrophorum* is sensitive to metronidazole, clindamycin, and imipenem, and less likely to be sensitive to erythromycin and penicillin.^[21]^ The patient did not achieve negative blood culture conversion and felt disoriented after treatment with piperacillin/tazobactam and clindamycin combination therapy for 5 days. An improvement was noted after treatment with meropenem, and this antibiotic regimen was maintained throughout the treatment period. There is no consensus on the optimal duration of antibiotic treatment; duration is commonly decided according to disease severity and clinical manifestations. The patient was treated for 4 weeks and studies have reported similar durations, with average lengths of treatment of 3 to 5 weeks.^[16,22]^

## 4. Conclusion

Lemierre syndrome is a rare disease and may start as a mild disease, but fatal complications can develop. Appropriate sepsis management and active complication control with a multidisciplinary approach could reduce the posttreatment sequelae for the patient.

## Author contributions

**Conceptualization:** Tae Hun Kim, Yong Shik Kwon, Jae Seok Park, Sun Hyo Park.

**Data curation:** Tae Hun Kim.

**Formal analysis:** Tae Hun Kim, Hyun Jung Kim, Sun Hyo Park.

**Investigation:** Tae Hun Kim.

**Methodology:** Tae Hun Kim, Mi-Ae Kim.

**Writing—original draft:** Tae Hun Kim, Seong Hwan Youn.

**Writing—review & editing:** Tae Hun Kim, Seong Hwan Youn, Mi-Ae Kim, Hyun Jung Kim, Yong Shik Kwon, Jae Seok Park, Sun Hyo Park.
